# Longitudinal changes in type 2 and *Aspergillus*-specific biomarkers during omalizumab therapy for allergic bronchopulmonary aspergillosis: a 36-week study in Chinese patients

**DOI:** 10.3389/fimmu.2026.1936285

**Published:** 2026-10-26

**Authors:** Peize Liu, Mengwei Wu, Xu Li, Xiao Xu, Haoyuan Li, Xueli Dong, Yanmeng Kang, Jianxue Bai, Chao Wang, He Geng, Ling Zhou, Yan Sun, Na Zhang, Yunqing Zhang, Na Liu, Tingting Gao, Liang Dong, Fen Liu, Lili Zhi

**Affiliations:** 1 Department of Respiratory, The First Affiliated Hospital of Shandong First Medical University & Shandong Provincial Qianfoshan Hospital, Shandong Institute of Respiratory Diseases, Jinan, China; 2 Department of Allergy, The First Affiliated Hospital of Shandong First Medical University, Shandong Provincial Qianfoshan Hospital, Shandong Institute of Respiratory Diseases, Jinan, China; 3 Department of Pediatrics, Huantai County Traditional Chinese Medicine Hospital, Zibo, China; 4 Department of Dermatology and Allergy, Huantai County Traditional Chinese Medicine Hospital, Zibo, China

**Keywords:** allergic bronchopulmonary aspergillosis, biomarker, eosinophils, FeNO, omalizumab, TARC, type 2 inflammation

## Abstract

**Background:**

Allergic bronchopulmonary aspergillosis (ABPA) is a severe type 2 inflammatory airway disease caused by hypersensitivity to *Aspergillus fumigatus*. Although omalizumab has been used as an adjunctive therapy for ABPA, longitudinal changes in type 2 inflammatory and *Aspergillus*-specific biomarkers during treatment remain incompletely characterized.

**Objective:**

To evaluate clinical outcomes and longitudinal biomarker changes during omalizumab therapy in patients with ABPA.

**Methods:**

We retrospectively studied 15 adults with ABPA treated with omalizumab 600 mg every 4 weeks and followed for 36 weeks. Clinical outcomes, including pulmonary function, fractional exhaled nitric oxide (FeNO), blood eosinophil counts, and oral corticosteroid (OCS) use, were assessed at baseline, week 12, and week 36. Serial measurements of total IgE, *A. fumigatus*-specific IgE and IgG4, thymus and activation-regulated chemokine (TARC/CCL17), IL-4, IL-5, and IL-13 were performed. Longitudinal changes were analyzed using the Friedman test, with false discovery rate (FDR)-adjusted q values calculated for multiple testing.

**Results:**

Omalizumab treatment was associated with reductions in OCS dose, FeNO, blood eosinophils, TARC, IL-4, IL-5, and IL-13 (all overall P<0.001, FDR q<0.001). Median OCS dose decreased from 15.0 to 0 mg/day, FeNO from 43.0 to 22.0 ppb, and eosinophils from 0.58 to 0.06 ×10^9^/L by week 36. Individual trajectories were predominantly downward, with monotonic declines in 80.0%–100% of participants depending on the marker. PEF increased significantly, whereas the increase in FEV1 was not statistically significant. *A. fumigatus*-specific IgE and IgG4 declined, while total IgE showed no sustained decrease. No significant associations were detected between total IgE and TARC or the measured cytokines (all nominal P≥0.062; all FDR q≥0.482). No serious treatment-related adverse events were observed.

**Conclusion:**

In this real-world ABPA cohort, omalizumab therapy was associated with corticosteroid reduction and parallel declines in several type 2-associated biomarkers over 36 weeks. These longitudinal clinical and biomarker profiles provide a more detailed picture of treatment-associated changes and a basis for prospective evaluation of their clinical relevance.

## Introduction

Allergic bronchopulmonary aspergillosis (ABPA) is a chronic inflammatory disease of the airways caused by a hypersensitivity reaction to *Aspergillus fumigatus*. It usually occurs in patients with asthma or cystic fibrosis and can lead to repeated exacerbations, mucus plugging, and progressive structural lung damage such as bronchiectasis if not treated in time. Although the condition has been recognized for decades, it still presents diagnostic and therapeutic challenges in daily practice (1, 2). Many patients are misdiagnosed as having uncontrolled asthma or recurrent pneumonia before ABPA is confirmed. By the time the diagnosis is made, some already have irreversible lung changes and impaired quality of life (3).

The cornerstone of therapy has long been systemic corticosteroids. Steroids are effective for acute control, but prolonged use brings well-known complications. In addition, a considerable proportion of patients relapse once steroids are tapered or stopped, creating a pattern of dependency. Antifungal drugs such as itraconazole and voriconazole have been added to reduce fungal load and decrease immune stimulation. They can be useful in some patients but are limited by side effects, drug interactions, and the emergence of resistance. Taken together, these problems mean that a significant group of patients with ABPA remain inadequately controlled or suffer long-term toxicity from their treatments (4). Over the past decade, biologic therapies have opened a new path for ABPA management (5–8). Among them, omalizumab, a recombinant monoclonal antibody against IgE, has attracted particular interest (9). Omalizumab was first approved for severe allergic asthma and later for chronic urticaria, and its ability to bind free IgE and block FcϵRI receptor activation makes it relevant for diseases driven by IgE-mediated hypersensitivity (10, 11). Several case reports and small studies have suggested that omalizumab can reduce exacerbations, improve symptoms, and allow tapering of corticosteroids in ABPA (12–15). Patients treated with omalizumab often describe fewer episodes of wheeze and cough, less sputum plugging, and a general improvement in control. Safety data so far are reassuring, with only mild local reactions reported in most cases.

However, the biomarker changes accompanying these clinical outcomes remain incompletely characterized. Published ABPA studies have emphasized exacerbations, lung function, and corticosteroid use, with more limited and heterogeneous longitudinal biomarker data (14, 16–19). Assessing inflammatory markers and *Aspergillus*-specific antibodies alongside clinical outcomes may help describe their differing trajectories during anti-IgE therapy. We therefore examined these measures at baseline, week 12, and week 36 in Chinese patients receiving omalizumab, considering both cohort-level trends and individual-patient changes.

## Methods

### Study design and setting

This retrospective real-world cohort study was conducted at the Department of Allergy and the Department of Respiratory, The First Affiliated Hospital of Shandong First Medical University (Shandong Provincial Qianfoshan Hospital), Jinan, China, a tertiary referral center for allergic and respiratory diseases. Consecutive patients with allergic bronchopulmonary aspergillosis (ABPA) who received omalizumab therapy between October 2024 and October 2025 were identified through electronic medical records. The primary objective of the study was to evaluate longitudinal clinical and immunological responses to omalizumab in patients with ABPA, with particular emphasis on dynamic changes in type 2 inflammatory biomarkers and *Aspergillus*-specific immune markers during treatment. The study was approved by the Institutional Review Board of The First Affiliated Hospital of Shandong First Medical University and was conducted in accordance with the Declaration of Helsinki. Written informed consent was obtained from all participants for treatment and use of anonymized clinical data for research purposes.

### Patient selection

Adult patients (≥18 years) diagnosed with ABPA according to the revised International Society for Human and Animal Mycology (ISHAM) criteria were eligible for inclusion (4). Diagnosis was established based on compatible clinical manifestations, evidence of *Aspergillus fumigatus* sensitization, elevated total serum IgE levels, and supporting radiological and/or immunological findings. Patients were included if they received omalizumab because of corticosteroid dependence, recurrent disease exacerbations, inadequate disease control despite conventional therapy, or intolerance to systemic corticosteroids and/or antifungal agents. Exclusion criteria included active pulmonary tuberculosis, non-tuberculous mycobacterial infection, uncontrolled malignancy, previous biologic therapy within 12 months before omalizumab initiation, or incomplete follow-up data. A total of 15 patients were included in the final analysis. Demographic characteristics, disease duration, comorbidities, radiological findings, previous treatments, and laboratory parameters were collected from medical records.

### Treatment protocol

All patients received omalizumab (Enyitan^®^, CSPC Pharmaceutical Group Limited, China) at a fixed dose of 600 mg administered subcutaneously every four weeks. This regimen was selected because baseline total serum IgE concentrations in all patients substantially exceeded the upper range of the standard dosing table approved for severe allergic asthma. Background therapies, including inhaled corticosteroids (ICS), long-acting β2-agonists (LABA), oral corticosteroids (OCS), leukotriene receptor antagonists, and antifungal agents, were continued or adjusted according to clinical response and physician judgment. OCS doses were gradually tapered whenever disease control permitted.

### Clinical and immunological assessments

Clinical and laboratory assessments were performed at baseline (week 0), week 12, and week 36 after initiation of omalizumab therapy. Clinical outcomes included pulmonary function parameters, fractional exhaled nitric oxide (FeNO), peripheral blood eosinophil counts and oral corticosteroid dosage. Pulmonary function testing included forced expiratory volume in one second (FEV1) and peak expiratory flow (PEF). Serial measurements included total serum IgE, *Aspergillus fumigatus*-specific IgE (m3-sIgE), *A. fumigatus*-specific IgG4 (sIgG4), thymus and activation-regulated chemokine (TARC/CCL17), IL-4, IL-5, and IL-13. Serum TARC, IL-4, IL-5, and IL-13 concentrations were each measured using conventional enzyme-linked immunosorbent assay (ELISA) kits from Thermo Fisher Scientific (Waltham, MA, USA), according to the manufacturer’s instructions; no multiplex assay was used. Safety outcomes included adverse events, injection-site reactions, treatment discontinuation, and serious adverse events throughout the study period.

### Statistical analysis

Continuous variables are presented as mean ± standard deviation or median with interquartile range (IQR), as appropriate, whereas categorical variables are presented as counts and percentages. Longitudinal changes across baseline, week 12, and week 36 were evaluated using the Friedman test, followed by Dunn’s multiple-comparison test for pairwise comparisons when appropriate. For the 12 longitudinal outcomes in **Table 1**, Benjamini–Hochberg FDR-adjusted q values were used to account for multiple testing, with q<0.05 considered statistically significant. Pairwise post hoc comparisons were interpreted using Dunn-adjusted P values. Categorical variables were compared using Fisher’s exact test. All statistical analyses were performed using GraphPad Prism version 10.0 (GraphPad Software, San Diego, CA, USA). Two-sided P values <0.05 were considered statistically significant, with FDR q values used to support interpretation of longitudinal findings. P and q values are reported as exact values unless <0.001. Patient-level trajectories were summarized as the number and percentage of participants whose week-36 value changed in the same direction as the cohort median relative to baseline. A monotonic trajectory was defined as movement in the cohort-level direction without reversal across baseline, week 12, and week 36; ties were permitted. Spearman rank correlations examined associations of total IgE with TARC, IL-4, IL-5, and IL-13 at each visit and associations between their baseline-to-week-36 changes. Benjamini-Hochberg FDR correction was applied across the 16 correlation tests.

**Table 1:** Longitudinal changes in clinical parameters, type 2-associated biomarkers, and *Aspergillus*-specific immune responses during omalizumab therapy.

Variable	Baseline	Week 12	Week 36	Overall *P*	FDR q
Clinical outcomes and pulmonary function					
FEV1 (% predicted)	68.0 (64.5–73.5)	71.0 (68.0–74.5)	72.0 (68.0–77.5)	0.138	0.138
PEF (% predicted)	72.0 (69.5–74.5)	76.0 (72.0–77.5)	76.0 (73.5–80.5)	<0.001	<0.001
FeNO (ppb)	43.0 (35.0–55.5)	30.0 (25.5–35.0)	22.0 (20.5–25.0)	<0.001	<0.001
OCS dose (mg/day)	15.0 (12.5–17.5)	7.5 (5.0–11.3)	0 (0–2.5)	<0.001	<0.001
Type 2-associated biomarkers					
Eosinophils (×10^9^/L)	0.58 (0.40–0.61)	0.30 (0.17–0.33)	0.06 (0.02–0.10)	<0.001	<0.001
TARC (pg/mL)	1443.4 (1288.5–1566.6)	997.8 (782.7–1154.4)	723.1 (551.4–799.1)	<0.001	<0.001
IL-4 (pg/mL)	5.67 (5.06–6.80)	4.10 (3.29–4.84)	2.23 (1.81–2.98)	<0.001	<0.001
IL-5 (pg/mL)	10.43 (8.75–12.08)	8.41 (7.45–10.06)	6.21 (4.41–6.55)	<0.001	<0.001
IL-13 (pg/mL)	9.77 (9.19–11.18)	7.82 (7.41–8.27)	5.87 (5.26–6.50)	<0.001	<0.001
*Aspergillus*-specific biomarkers					
Total IgE (kU/L)	1112.0 (1008.5–1349.0)	1165.5 (1066.0–1407.5)	1160.0 (1046.3–1362.3)	<0.001	<0.001
*A. fumigatus*-specific IgE (kUA/L)	19.6 (10.0–29.7)	18.7 (10.1–30.6)	18.5 (9.7–27.5)	0.005	0.006
*A. fumigatus*-specific IgG4 (mgA/L)	385.2 (267.9–468.3)	330.4 (223.4–409.8)	320.4 (209.2–396.5)	<0.001	<0.001

Data are presented as median (IQR). Longitudinal assessments were performed at baseline, week 12, and week 36 following initiation of omalizumab therapy. Overall differences among the three time points were evaluated using the Friedman test, followed by Dunn’s multiple-comparison test for pairwise analyses. FeNO, fractional exhaled nitric oxide; OCS, oral corticosteroid; EOS, eosinophils; TARC, thymus and activation-regulated chemokine (CCL17); FEV1, forced expiratory volume in one second; PEF, peak expiratory flow.

## Results

### Baseline characteristics

A total of 15 patients with ABPA who received omalizumab therapy were included in the study. Baseline demographic and clinical characteristics are summarized in **Table 2**. The median age was 38.0 years (IQR, 35.0–41.5), and 11 patients (73.3%) were male. All patients had concomitant asthma, and four patients (26.7%) had bronchiectasis. Nine patients (60.0%) were never smokers and six patients (40.0%) were former smokers. The median duration of ABPA was 5.0 years (IQR, 4.0–6.5). Because baseline total serum IgE levels substantially exceeded the upper range of the approved asthma dosing table, all patients received omalizumab 600 mg every four weeks throughout the study period.

**Table 2:** Baseline characteristics of the 15 patients with allergic bronchopulmonary aspergillosis (ABPA) treated with omalizumab.

Number of patients	N=15
Age, years	38.0 (35.0-41.5)
Male sex, n (%)	11 (73.3%)
Female sex, n (%)	4 (26.7%)
Asthma, n (%)	15 (100%)
Bronchiectasis, n (%)	4 (26.7%)
Never smokers, n (%)	9 (60.0%)
Former smokers, n (%)	6 (40.0%)
ABPA duration, years	5.0 (4.0-6.5)
Omalizumab regimen	600 mg every 4 weeks
Baseline total IgE, kU/L	1112.0 (1008.5-1349.0)
Baseline eosinophils, ×10⁹/L	0.58 (0.40-0.61)
Baseline FeNO, ppb	43.0 (35.0-55.5)
Baseline FEV1, % predicted	68.0 (64.5-73.5)
Baseline PEF, % predicted	72.0 (69.5-74.5)
Baseline OCS dose, mg/day	15.0 (12.5-17.5)

Data are presented as median (interquartile range) for continuous variables and number (percentage) for categorical variables, unless otherwise indicated. All patients fulfilled the International Society for Human and Animal Mycology (ISHAM) diagnostic criteria for ABPA and received omalizumab 600 mg every 4 weeks. ABPA, allergic bronchopulmonary aspergillosis; FeNO, fractional exhaled nitric oxide; FEV1, forced expiratory volume in one second; IgE, immunoglobulin E; OCS, oral corticosteroid; PEF, peak expiratory flow.

### Clinical outcomes and pulmonary function

Clinical and laboratory parameters at baseline, week 12, and week 36 are summarized in **Table 1**. Following initiation of omalizumab, improvements were observed in several disease-related clinical outcomes. Median oral corticosteroid (OCS) dose decreased from 15.0 mg/day (IQR, 12.5–17.5) at baseline to 7.5 mg/day (5.0–11.3) at week 12 and 0 mg/day (0–2.5) at week 36, with a significant overall longitudinal change after FDR correction (overall P<0.001, FDR q<0.001). FeNO levels also declined from 43.0 ppb (35.0–55.5) at baseline to 30.0 ppb (25.5–35.0) at week 12 and 22.0 ppb (20.5–25.0) at week 36 (overall P<0.001, FDR q<0.001). Pulmonary function showed a heterogeneous pattern. Median PEF increased from 72.0% predicted (69.5–74.5) at baseline to 76.0% predicted (72.0–77.5) at week 12 and remained at 76.0% predicted (73.5–80.5) at week 36 (overall P<0.001, FDR q<0.001). In contrast, FEV1 showed only a modest numerical increase, from 68.0% predicted (64.5–73.5) at baseline to 71.0% predicted (68.0–74.5) at week 12 and 72.0% predicted (68.0–77.5) at week 36, without a statistically significant overall difference (overall P = 0.138, FDR q=0.138).

### Longitudinal changes in type 2-associated biomarkers

Circulating type 2-associated biomarkers declined during follow-up (**Table 1**, **Figure 1**). Median blood eosinophil counts decreased from 0.58 × 10^9^/L at baseline to 0.30 × 10^9^/L at week 12 and 0.06 × 10^9^/L at week 36. Median TARC concentrations decreased from 1443.4 to 997.8 and 723.1 pg/mL, respectively. Over the same time points, median IL-4 levels decreased from 5.67 to 4.10 and 2.23 pg/mL, IL-5 from 10.43 to 8.41 and 6.21 pg/mL, and IL-13 from 9.77 to 7.82 and 5.87 pg/mL. All five biomarkers showed significant overall longitudinal changes after FDR correction (all P < 0.001 and q < 0.001). At the individual level, week-36 values of FeNO, eosinophils, TARC, IL-4, IL-5, and IL-13 were lower than baseline in all 15 participants. However, declines were not uniformly monotonic. Monotonic declines across both intervals occurred in 13 (86.7%), 14 (93.3%), 15 (100%), 12 (80.0%), 14 (93.3%), and 12 (80.0%) participants, respectively. For IL-13, three participants showed an increase during one interval, but their week-36 values remained below baseline.

**Figure 1:**
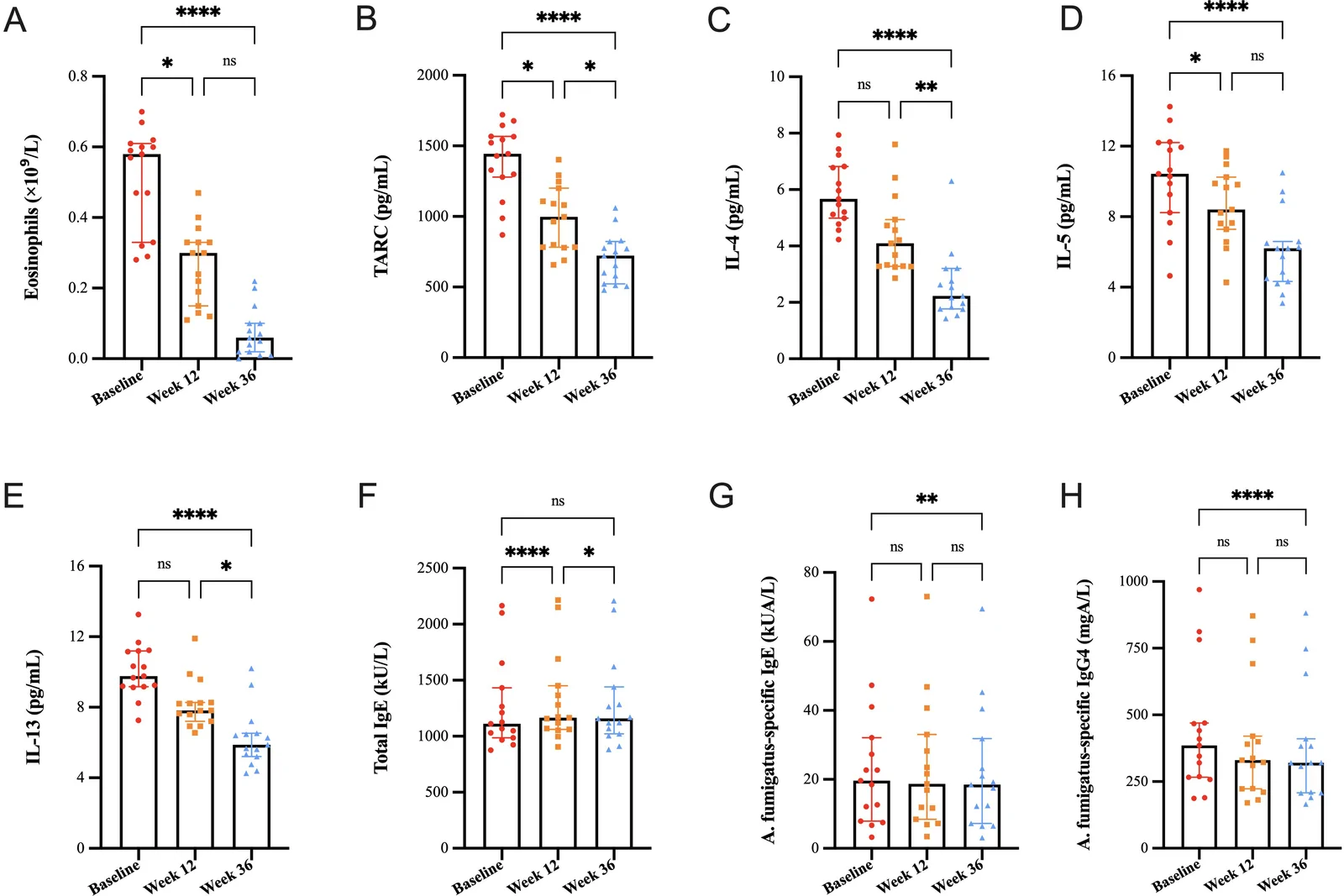
Biomarker changes during omalizumab therapy. **(A)** Eosinophils, **(B)** TARC, **(C)** IL-4, **(D)** IL-5, **(E)** IL-13, **(F)** total IgE, **(G)** A. fumigatus-specific IgE, and **(H)** A. fumigatus-specific IgG4 in 15 patients. Red circles, orange squares, and blue triangles represent baseline, week 12, and week 36, respectively. Symbols show individual observations; bars and error bars indicate medians and IQRs. Pairwise comparisons were performed using Dunn’s multiple-comparison test following the Friedman test. Symbols indicate Dunn-adjusted pairwise P values; FDR-adjusted q values for the overall longitudinal tests are reported in **Table 1**. *P < 0.05, **P < 0.01, ***P < 0.001, ****P < 0.0001; ns, not significant. TARC, thymus and activation-regulated chemokine.

### Longitudinal changes in *Aspergillus*-specific biomarkers

Changes in *Aspergillus*-related biomarkers differed from those observed for type 2 inflammatory markers. Median total IgE levels increased from 1112.0 kU/L (1008.5–1349.0) at baseline to 1165.5 kU/L (1066.0–1407.5) at week 12 and remained at 1160.0 kU/L (1046.3–1362.3) at week 36, with a significant overall longitudinal difference (overall P<0.001, FDR q<0.001). In contrast, *Aspergillus fumigatus*-specific IgG4 decreased progressively during treatment, from 385.2 mgA/L (267.9–468.3) at baseline to 330.4 mgA/L (223.4–409.8) at week 12 and 320.4 mgA/L (209.2–396.5) at week 36 (overall P<0.001, FDR q<0.001). *A. fumigatus*-specific IgE showed a more modest decline, from 19.6 kUA/L (10.0–29.7) at baseline to 18.7 kUA/L (10.1–30.6) at week 12 and 18.5 kUA/L (9.7–27.5) at week 36, but the overall change remained significant after FDR correction (overall P = 0.005, FDR q=0.006). Spearman analyses showed no statistically significant association between total IgE and TARC, IL-4, IL-5, or IL-13 at baseline, week 12, or week 36 (all nominal P≥0.062). Baseline-to-week-36 changes in these markers also showed no statistically significant correlations with the corresponding change in total IgE (all P≥0.143). After correction across all 16 tests, all FDR q values were ≥0.482 (**Table 3**).

**Table 3:** Spearman correlations of type 2-associated markers with total IgE.

Marker	Baseline rho (P)	Week 12 rho (P)	Week 36 rho (P)	Change rho (P)
TARC	0.36 (0.191)	0.38 (0.160)	0.27 (0.334)	0.04 (0.894)
IL-4	-0.23 (0.405)	-0.12 (0.657)	0.34 (0.211)	-0.40 (0.143)
IL-5	-0.49 (0.062)	-0.29 (0.302)	0.06 (0.820)	0.12 (0.671)
IL-13	0.37 (0.177)	-0.35 (0.201)	-0.31 (0.254)	-0.11 (0.708)

Values are Spearman rho with nominal two-sided P values in parentheses. Change denotes week 36 minus baseline. Benjamini-Hochberg correction was applied across all 16 tests; all FDR q values were ≥0.482.

## Discussion

In this 36-week real-world cohort of Chinese patients with ABPA, omalizumab therapy was associated with a reduction in corticosteroid use, higher PEF, and parallel decreases in several type 2-associated biomarkers. The median daily OCS dose fell from 15 mg at baseline to 0 mg at week 36, consistent with the corticosteroid-sparing outcomes reported in systematic reviews and longitudinal ABPA studies (5, 12, 17, 18). The present study adds serial measurements of TARC, IL-4, IL-5, IL-13, and *Aspergillus*-specific antibodies alongside clinical outcomes, providing a detailed account of treatment-associated changes over three assessments. The parallel declines in FeNO, eosinophils, TARC, IL-4, IL-5, and IL-13 were a notable feature of this cohort. These changes occurred alongside OCS tapering, and individual profiles showed that the downward trends were not confined to cohort medians: depending on the marker, 80.0%–100% of participants had monotonic declines across both intervals. The remaining trajectories illustrate within-patient variation that would be obscured by group summaries alone. Sah et al. reported that, despite reductions in cohort medians after oral prednisolone treatment, periostin and carcinoembryonic antigen increased in 34% and 30% of patients, respectively, highlighting the limitations of group summaries for individual monitoring (20). Omalizumab targets free IgE, and attenuation of IgE-mediated mast-cell and basophil signaling offers a plausible explanation for downstream biomarker changes (21, 22). TARC, which recruits CCR4-positive Th2 lymphocytes, provides an additional type 2-associated measure alongside eosinophils and cytokines (23–25). Together, these observations extend the longitudinal description of type 2-associated biomarker profiles during omalizumab treatment.

Total serum IgE followed a different trajectory, increasing initially and remaining near baseline at week 36. This contrasts with the pooled reduction reported in a meta-analysis (5). Cai et al. found no significant overall reduction in total IgE and reported different patterns according to concomitant allergic rhinitis (18). Total and free IgE are not equivalent, and omalizumab–IgE complex formation can complicate interpretation of measured total IgE during treatment (21, 22). No significant correlations were detected between total IgE and TARC or the measured cytokines, either at individual visits or for their baseline-to-week-36 changes. Larger cohorts will help clarify the relationships between these measures and the additional information that each may provide alongside clinical assessment. Longitudinal assessment of *A. fumigatus*-specific antibodies adds another dimension to these findings. Specific IgG4 decreased overall and in 13 of 15 participants, whereas the decline in specific IgE was more modest. The distinction between specific IgG4 and total specific IgG is important: previous work has questioned the value of total *A. fumigatus*-specific IgG for monitoring treatment response (26). Specific IgG4 may vary with the intensity and duration of fungal antigen exposure (27), making changes in antigenic stimulation or background therapy possible explanations for the observed pattern. Because omalizumab does not directly target IgG4, the decline should be considered an exploratory serological observation. Its relationship to antigen exposure and clinical outcomes merits prospective investigation.

PEF improved during follow-up, whereas the increase in FEV1 was not statistically significant. Voskamp et al. reported fewer exacerbations and lower FeNO with omalizumab (19), whereas Cai et al. also reported significant FEV1 improvement (18), which was not observed in our cohort. Baseline obstruction and structural airway changes may help explain this variability. Omalizumab was generally well tolerated during the 36-week observation period: no serious treatment-related adverse events, treatment discontinuations, or anaphylactic reactions were recorded, and three patients experienced self-limited injection-site reactions. The study’s retrospective, single-center design and sample size limit generalizability and causal inference. Without a control group, the effects of omalizumab cannot be separated from OCS tapering and other treatment adjustments. The absence of nonresponders precluded assessment of predictive or routine-monitoring utility. Th2 and ILC2 phenotyping, type 1 markers, broader cytokine/growth-factor profiling, and free IgE were unavailable, limiting mechanistic interpretation. These data therefore characterize measured biomarker changes rather than direct cellular or type 1 immune effects. The correlation analyses were exploratory, with limited power to detect associations after multiple-testing correction, and longer follow-up is needed to assess relapse. Prospective studies incorporating a wider range of clinical responses and more detailed immune profiling can build on the longitudinal patterns identified here.

## Conclusion

In Chinese patients with ABPA, 36 weeks of omalizumab therapy was associated with corticosteroid reduction, improved PEF, and parallel declines in several type 2-associated biomarkers. The combined clinical, inflammatory, and *Aspergillus*-specific antibody profiles add longitudinal evidence to the characterization of treatment-associated changes. These findings provide a basis for prospective studies to clarify the clinical relevance of individual biomarker trajectories.

## Data Availability

The original contributions presented in this study are included in the article. Further inquiries can be directed to the corresponding author.
